# Formalizing Permission to Delegate and Delegation with Policy Interaction

**DOI:** 10.3390/s25164915

**Published:** 2025-08-08

**Authors:** Azan Hamad Alkhorem, Daniel Conte de Leon, Ananth A. Jillepalli, Jia Song

**Affiliations:** 1 Department of Computer Engineering, College of Computer Science and Information Technology, Majmaah University, Al Majmaah 11952, Saudi Arabia; ah.alkhorem@mu.edu.sa; 2 Department of Computer Science, University of Idaho, Moscow, ID 83844, USA; dcontedeleon@ieee.org (D.C.d.L.); jsong@uidaho.edu (J.S.); 3 College of Electrical Engineering and Computer Science, Washington State University, Pullman, WA 99164, USA

**Keywords:** access control models, permission, permission to delegate, delegation, redundant, conflict detection

## Abstract

In the context of Internet of Things (IoT) intelligent systems, the latest research regarding delegation using an access control model has gained attention, reflecting the need for models to support more functionalities in relation to hierarchical delegation. With respect to delegation procedures within access control, issues arise after delegation concerning the permissions to others with respect to revocation. Redundancy and conflict arising from delegation can occur depending on the delegation policies used within the hierarchical structure. This article discusses implementation of positive delegation represented by “YES” and negative delegation represented by “NO”. Furthermore, we also consider permission to delegate positively and negatively represented by (YES and NO). These challenges are addressed by creating additional features in a hierarchical policy model (HPol). The implementation was created using Python (ver. 3.10) code to verify the advantages of the approach, through experimentation under different scenarios. The model also has the capability to manage and adapt features of the Internet of Things (IoT) to a blockchain architecture, enhancing security and verification during the delegation process and increasing the scalability of Internet of Things (IoT) intelligent environment systems.

## 1. Introduction

Access control as well as decentralization play important roles in the management of IoT and blockchain systems. Moreover, they are becoming increasingly necessary as intelligent systems evolve [1]. Blockchain systems have been defined in several prior works [2,3]. The traditional access control model used relies on centralized trust, which can be problematic [4]. Decentralization of blockchain technology can provide a useful framework for controlling access to resources and enabling trust by the creation of delegation rules [5]. Delegation control methods using blockchain technology can support accessible revocation and secure delegation, which is especially important in an intelligent system environment [6].

Delegation through access control defines permissions that a user (or subject) can grant to delegate tasks to other users or groups [7]. A delegator may delegate any action already set up to a delegatee and the delegator will then lose the ability to take that action [8]. ‘Permission to delegate’ can be defined as a preset policy before the delegation is requested [9]. This approach to permission to delegate provides a more effective way of verifying the delegation before it is permitted or transferred between users [10]. This approach can support access control mechanisms in IoT applications in domains such as the Industrial IoT, drone swarms, and the Internet of Vehicles [11].

### 1.1. State of the Art

Numerous scholars have researched creating models for access control. Some of these models have been improved to include delegation functionalities. The issues presented in Table 1 include the detailed problems facing access control models with respect to delegation capabilities. A systematic literature review was conducted by [12], the results of which are summarized in Table 1. The review revealed that while many models include access control policies and a basic form of delegation, their provision for critical functionalities, including permission to delegate, redundancy, and conflict detection, both within standard policies and between delegation policies, and across diverse policy types, covering both positive and negative aspects, along with separation of duties, remains quite limited. Although a few studies touch upon specific functionalities covered in the analysis, no currently identified access control model demonstrates the capability to comprehensively support all functionalities.

### 1.2. The Problem

Based on the insights obtained from the articles referred to in Table 1, it is clear that organizations require a flexible access control model to manage a variety of policies and delegation methods. As organizations increasingly adopt zero-trust [13] policies for reducing risks and enhancing security, there is a critical need for any access control model to be adaptable. Such a model should, at the very least, encompass the functionalities outlined in Table 1, including different policy types, such as positive and negative permissions to delegate, and both types of delegation. Characteristics of delegation are listed in Table 2. Current access control models also tend to be inadequate to address IoT-specific challenges, such as scalability, decentralization, constrained devices, and fine-grained delegation. The work proposed in this manuscript seeks to address the delegation limitations of models in detail.

### 1.3. Contributions

The contributions of this research are as follows:

1. We incorporate full support for permission to delegate policies in the HPol model. This consists of (a) positive and (b) negative permission to delegate. Details regarding this contribution can be found in Section 3.3.
2. We implement comprehensive support for delegation in the HPol model, covering both (a) positive and (b) negative delegation. This contribution is also detailed in Section 3.3.
3. For each outlined contribution, we refine the HPol formal model and develop relevant Python code.
4. We conduct thorough examinations of scenarios involving model requests and responses through detailed experiments, with comprehensive explanations provided in the dedicated sections of this article.

The rest of this article is structured as follows: Section 2 provides the relevant background for the article and describe related work on access control delegation; Section 3.1 describes our previous work; Section 3.3 present the specific contributions of the article. Section 4 and Section 5 conclude the article and consider potential areas for future exploration.

## 2. Background and Related Work

### 2.1. Access Control

Access control is essential for information and device security, preventing unauthorized access and regulating user privileges. This section explores various models, including attribute-based, discretionary, mandatory, and role-based access control, positive and negative policies, positive and negative permission to delegate, and positive and negative delegation.

In the field of cybersecurity, access control is playing a growing role, enabling authorized users to access system resources while blocking unauthorized access. Specifically, attribute-based access control (ABAC) and role-based access control (RBAC) stand out. ABAC relies on user and object attributes, while RBAC restricts access based on organizational roles. These models offer diverse approaches to access control.

### 2.2. Access Control Models

*Attribute-based access control model (ABAC):* ABAC is a type of system that simplifies access control by focusing on user and object attributes, environmental factors, and rules, rather than standard ownership and roles. ABAC prioritizes user identity and classifies attributes into domains, including user, object, environmental, connection, and administrative attributes, defining access requirements. Critical ABAC components include users, objects, attributes, policies, and their connections, formalized in various ABAC models. For further details about the distribution of ABAC please refer to the research conducted by [14].

*Discretionary access control (DAC):* DAC functions as an access control system in which the policies governing access to secured computers, records, or resources are established by administrators or owners. These policies outline the eligibility criteria for resource access and often grant administrators the ability to limit the spread of access permissions. A common criticism of DAC models concerns the absence of centralized authority control. To learn more about DAC concepts, please refer to the [15] survey.

*Mandatory access control (MAC):* MAC is a security model whereby a central entity oversees access permissions, which are determined by different security stages. Public institutions and armed forces often employ a DAC model to control their operating systems, hardware resources, and security cores. MAC can approve or disapprove access to specific computer resources based on device or user information checks. For more information, please refer to the publication by [15].

*Role-based access control (RBAC):* RBAC is an alternative to DAC and MAC access controls, using a different approach to regulating access to computer resources. For additional details on RBAC systems, please see the survey reported in [15].

### 2.3. Positive and Negative Permission to Delegate

Positive permission to delegate (YES) indicates that any user or multiple users have permission to delegate, meaning that they can allow the delegation. Negative permission to delegate (NO), on the other hand, indicates that any user or multiple users can prohibit the delegation from being granted. Both of these types of permission to delegate are created prior to the delegation request.

### 2.4. Positive and Negative Delegation

A positive delegation policy (YES) allows a user to delegate his or her right to perform tasks or duties to others in some circumstances. On the other hand, a negative delegation (NO) policy specifies that a user can refuse to delegate an action to others. More information about delegation with respect to access control model features can be found in [16,17], which describes delegation models with different features.

### 2.5. Related Work

Based on our earlier analysis and research classification survey [12] which provides a comparison of previous studies related to access control attributes, we focus on issues highlighted in the research that need to be investigated and solved with respect to the features of access control models.

Table 1 summarizes previous research based on our survey results. The survey initially identified 172 articles from the literature. After deleting duplicates, there were 137 articles left. Following relevance analysis, 39 articles were identified as relevant. Out of the 39 publications considered relevant, four were surveys and 35 were individual studies. Subsequently, an analysis of knowledge gaps was undertaken.

We first consider features relating to supporting positive and negative policies plus redundancy and conflict detection highlighted in our previous study [18] (submitted under review). They represent solutions that have been successful, while the features highlighted in blue with a green check mark in Table 1 signify current work in this article. The check marks highlighted in blue, detailed in Section 3.3, denote support for both types of permission to delegate, and both types of delegation. Furthermore, we provide an overview of the 35 relevant articles identified in our survey [12].

According to [19], a delegation model based on socio-technical design, cooperation and collaboration theory best fits the needs of social networks. The model was created with ontologies and implemented with Facebook APIs. When multiple policies overlap, policy conflicts and redundant policies are identified. The authors investigate the model’s expressive power, and its acceptability with users is tested. It is additionally evaluated in terms of social validity by comparing to 27 previous delegation models based on socio-technical validity parameters derived from socially accepted principles.

Article [20] describes a new delegation approach for extended role-based access control models, showing that it is adaptable for dealing with administration and delegation requirements in a united framework. The approach also makes it simple to express various delegation and revocation dimensions. Because the model is object-oriented, privileges are not directly adjusted; instead, objects with certain semantics can modify abilities such as permissions and constraints. Each object represents a power assignment or delegation, such as a user to a role or a user or a role to a permission. Limitations of the work include incompatibility of various types of permissions, whether positive or negative, for different types of delegation, such as positive and negative, and the conflict that exists between them.

The authors of [21] introduced a strategy for implementing a systematic and automated technique of determining if a subject has the authority to carry out delegation in a way that does not result in conflict or separation of duties prior to system enforcement. They used the Ponder language in their project on authorization and delegation policies. Ponder authorization policies are simply access control policies that are designed to prevent unauthorized resource access. A positive permission policy defines the actions that a subject is permitted to take on target objects. Negative authorization procedures that restrict individuals’ access to specified resources are not addressed since delegation of limitations is illogical. From our perspective, both positive and negative conflict policies must be evaluated rather than solely focusing on the positive while dismissing the conflict. Furthermore, it is important to consider how both kinds of permission to delegate conflict with one other, as well as with either positive or negative policy, or with both types of delegation. It is necessary to cover both positive and negative policy, both types of delegation authorization, and both types of delegation conflict. We discuss the separation of duties conflict and the violation of the separation of duties rule further below.

To empower accurate specification and verification of such policies, Ref. [22] proposes a model-driven engineering (MDE) approach based on UML and the Object Constraint Language (OCL). The authors begin by presenting a categorization of the different kinds of RBAC permission policies proposed in the literature. The authors propose the GemRBAC model, which is a generalized RBAC model that incorporates all of the entities needed to establish the classified policies. The model is a conceptual model that can function as a data model for data gathering and verification. The classified RBAC policies are formalized as OCL constraints on the GemRBAC model. To accommodate such operationalization, the authors utilize the Ecore version of the GemRBAC model and the OCL constraints corresponding to the categorized models publicly available online. Finally, SoD constraints are supported to avoid a mutual exclusion relation among roles, permissions, or users.

Ref. [23] presents a comprehensive delegation method for role-based access control based on the organization-based access control (OrBAC) formalism. This includes clearly establishing delegation limitations, such as the requisite settings for the user delegating and the user receiving the delegation, role-to-role and user-to-user, to delegate a permission. The model is more adaptable, simpler to operate, and more comprehensive. Moreover, the OrBAC model offers accommodations for dealing with delegation needs without the requirement of additional elements. The OrBAC model is built around multi-granular as well as contextual licensing requirements. This allows users to specify numerous delegation characteristic features, such as permanence, totality, revocation, and so on.

Ref. [24] presents a Cellular Automata (CA)-based conflict resolution approach for RBAC. The scalability of general computing environments requires role delegation in RBAC. This practical principle, however, can lead to conflicts during compile and operate situations. The authors propose a policy for SoD and demonstrate conflict-free role delegation. The delegation mechanism, which is based on a type of CA known as Multiple-Attractor CA (MACA), can be applied effectively as an authorizing approach in complicated automated machines within RBAC.

The authors of [25] map RBAC onto multiple-attractor cellular automata for easy management (MACA). The restriction on SoD and role delegations can be easily addressed in MACA using pseudo-exhaustive (PE) bit patterns. The authors also demonstrate how to resolve conflicts between specified SoD constraints and delegation operations using PE-bit patterns. Because multiple subjects can grant authorizations in RBAC, administration becomes more difficult to control, and cascading and cyclic permissions may occur. As a result, the authors create an efficient mapping and delegation methodology based on MACA and utilizing PE-bit patterns. The proposed model can effectively prevent unwanted user intentions while limiting delegation time and avoiding conflicts. As a result, the authors anticipate that their mechanism will be effective for approving a technique in a difficult and complicated environment within RBAC’s automatic system.

The authors of [26] developed an XML-based delegation and revocation (XDR) model for WISs, which supports fine-grained delegation and managed resource spreading. In addition, the proposed model allows for a variety of delegation and revocation methods, including single-step delegation, multi-step delegation, multiple delegation, partial delegation, separation of duties, and cascading revocation. A prototype was developed and tested to illustrate the potential of the proposed model.

Different models, including PBDM, RBDM0, and RDM2000, were compared and contrasted in [27]. Total and user-to-user delegation are handled by the RBDM0 and RDM2000 models. In terms of approval administration, the PBDM model allows delegation from role-to-role and user-to-user, in addition to delegation during the role and permission phases, giving it a wide range of options. The authors included some non-support constraints, like the SoD for role-to-role delegation and user-to-user delegation, in the PBDM model. A delegation model was developed based on permission attributes, allowing system administrators to successfully handle permission phase delegation and partial delegation as well as inheritance restrictions. In future work, the model will require to be enhanced to include other delegation features, such as responsibility delegation and negative delegation.

The research study conducted by [28] presents a logic-based framework for decentralized RBAC systems that allows for dynamic delegation. The framework provides the opportunity for an administrator to delegate responsibilities and permissions between roles. The idea of delegation trust is considered and the authors demonstrate how extended logical programming can be used to define roles and delegation responsibilities in terms of trust levels, delegation scope, role advantages and their distribution, dispute resolution, and the resolution of conflicts. Moreover, the systems proposed allow implementation of role restrictions, such as SoD, role structure, and defining the limitations of cardinality. The designed system is extensible and provides a solid platform for developing and implementing advanced role-based policies for access control within decentralized settings. Based on the administration’s delegation characteristics, the role access control is significantly enhanced. Another factor to consider is multiple delegation conflict, which can arise in either positive or negative delegation between numerous roles. Furthermore, both types of permission to delegate authority must be granted or rejected before delegation is approved between multiple roles.

In [29], the authors discuss user-to-user delegation issues by demonstrating how delegations are implemented in the RBAC model. The model is distinguished in giving the user complete power about which authority he or she wishes to delegate, as perceived differently in relation to delegation at the role point, where all privileges of such a role have to be delegated. Furthermore, delegation and revocation are addressed, with a comprehensive set of rules for further delegations in the model for actions related to a right, as well as generic restrictions for further delegation of direct authority. Additional plans will be required to provide a more comprehensive system for managing rights.

Authorization, which is an extremely vital aspect of RBDM, can create conflict in situations where a user grants permissions to delegated users while another user grants negative permissions. The authors of Ref. [30] propose using negative authorization in role-based group delegation to solve the conflict issue. The authors begin by presenting delegation models for granting and revocation, and then investigate the implications of negative delegation authorization on the hierarchy of roles.

Ref. [31] presents a delegation model based on a decentralized management role graph model that supports permission to user, user-to-user, and role-to-role delegation by combining users or tasks given and defining the user role task. In the delegation model, the model is divided into two parts: a static part allows for easier administration and deployment and a dynamic part is left for future work. The authors include details regarding a member’s or team’s modifications, role hierarchy processes, and RBAC operational processes that are relevant to specific delegation issues.

Ref. [32] considers how to clarify and enforce delegation permissions. The article describes a rule-based methodology for developing authorization policies. Using a rule-based language, the authors were able to determine and enforce permission restrictions in powerful and flexible ways. Furthermore, the rules can be used to identify potential future exceptions and handle potential conflicts. It is suggested that in large, distributed role-based systems, determining and adding complicated delegation authorization policies are difficult and time-consuming duties. Delegation is intended to meet user needs by specifying an authorization policy, whereas organizational policies can be stated to impose restrictions. The approach simplifies role administration in role-based systems through delegation.

## 3. Formal Models of Permission to Delegate and Delegation

### 3.1. Prior Work on the Hierarchical Policy Model HPol

Automating the validation and enforcement of high-level security standards, the HPol model transforms detailed security settings into a more advanced model. It determines resource access and usage permissions. Using Directed Acyclic Graphs (DAGs), it represents policies for subjects, actions, and objects. Policy paths through the graph nodes indicate permitted or prohibited policies. A detailed description can be found in Section 3.2.

### 3.2. Positive and Negative Policy in the HPol Model

**Figure A1:**
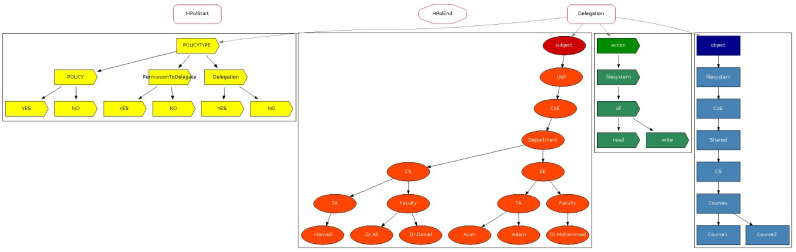
Example of HPol Model used throughout the rest of this article in each and all experiments. Note that this model shows the organizational structure but no polices (Empty Policy Model) considering policy, permission to delegate, and delegation.

We implement authorization using a new subgraph (DAG) and a distinct security policy method, regulating resource access before any actions. The HPol model employs a hierarchical policy shown in Figure A1, with nodes indicating positive “YES” or negative “NO” policies. This section focuses on the Policy node’s negative and positive policies, while PermissionToDelegate and Delegation nodes are discussed in Section 3.3.

**Figure A2:**
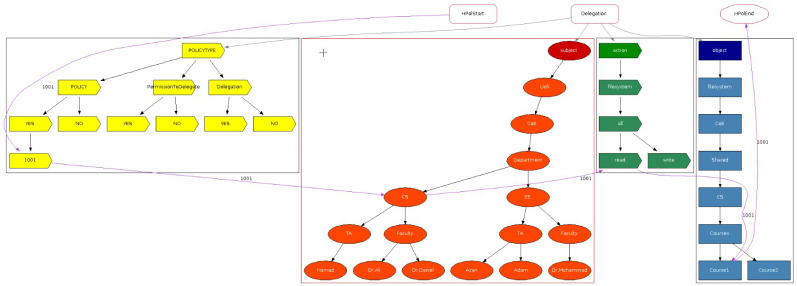
Resulting HPol model after requesting adding a positive policy to the empty HPol model example at the grandparent (subject) node. Yellow color represents policies. Orange color represents subjects. Green color represents action. Blue color represents objects.

Positive policies create a new node under “YES” with a unique PPID, while negative policies create a node under “NO” with their unique PPID. For example, in Figure A2, if a user is granted permission to read a specific object, they receive a unique PPID (1001).

**Figure A3:**
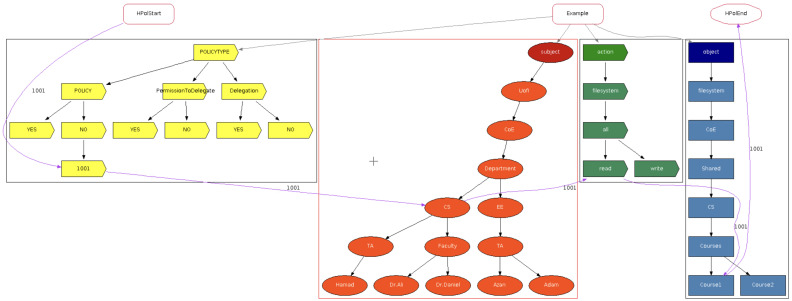
Resulting HPol model after requesting adding a negative policy to the empty HPol model example at the grandparent (subject) node. Yellow color represents policies. Orange color represents subjects. Green color represents action. Blue color represents objects.

Negative permissions are established within the “NO” node, which is directly linked to the POLICY node. When a user is denied permission, a distinctive PPID (1001) is assigned to them, as illustrated in Figure A3.

Simultaneously creating “YES”, which can include multiple YES policies, and “NO”, which can consist of multiple NO policies, allows for unique PPID distributions for each policy. This approach accommodates various policy combinations, including positive-positive (YES-YES), positive-negative (YES-NO), negative-negative (NO-NO), and negative-positive (NO-YES) policies. It is important to carefully manage potential conflicts, redundancies, and address partial policy considerations, as successfully resolved in our prior peer-reviewed publication [18].

Table 3 uses symbols: (-) for no authorization, “YES” for positive policies, and “NO” for negative policies. The (-) means no policy or authorization, “YES” grants access, and “NO” denies access. Table 4 and Table 5 detail case studies illustrating the progression from empty policies to positive and negative authorizations.

Figure A1 and Figure A2 illustrate the transformation of user permissions in the HPol model, progressing from empty policies to positive permits “YES”. In Figure A2, a user transitions from empty policies to positive permission “YES” in the HPol subgraph model, granting all subjects access to read Course1 under PPID (1001). The following is a description of the policy path direction of the positive policy “YES” request on the HPol model:

1. The HPol model begins at the HPolStart node and proceeds to the PPID (1001) node in the policy DAG, which is under the “YES” POLICY node. This indicates positive policy, as shown in Figure A2, which shows that CS’s users are the first and only unique PPID that has been created (1001).
2. Move from the PPID (1001) node to the “CS” node within the (Subject) DAG.
3. Move from the “CS” (Subject) node to the read (Action) node (DAG).
4. From the read (Action) node, move to the Course1 node in the Objects DAG.
5. The HPolEnd node, which must be connected, represents the endpoint of the HPol model. The last step is to move from the Course1 (Object) node to the HPolEnd node.

In Table 3, we show the transition from empty policies to positive ones for users, where positive policies are marked as “YES” with a unique PPID (1001). Table 6 explores cases involving permission delegation. The HPol model consistently follows a specific path from start to finish, as depicted in Figure A5 and Figure A6.

For example, in Figure A2, a positive policy is established for users in the “CS” node granting permission to read Course1. The path begins at HPolStart, creates a new node under PPID (1001), moves through Subject (DAG), Action (DAG), Object (DAG), and concludes at HPolEnd. These results are important for explaining permission delegation and delegation policy requests at a different level node in the HPol model, which will be discussed later.

Table 5 presents a case study with an empty policy (-) and a negative policy “NO” denying user access to an object’s action. Figure A3 introduces “NO” under (POLICY) node in the HPol model, creating PPID (1001) for negative policies. The HPol model’s policy path runs from HPolStart to HPolEnd, passing through PPID, user, action, and object, as illustrated in Figure A3. The following is a description of the policy path direction of the negative policy “NO” request on the HPol model:

1. The HPol model, beginning at the purple arrow’s HPolStart node and reaching PPID (1001) under the “NO” node in the POLICY node in Figure A3, shows a unique PPID has been assigned to CS’s users (1001).
2. From the PPID (1001) node, navigate to the “CS” (Subject) node within the Subject DAG.
3. Navigate from the “CS” (Subject) node to the read (Action) node in the Subject DAG.
4. In the Object DAG, go from the read (Action) node to the Course1 node.
5. The model’s endpoint is represented by the HPolEnd node, which must be connected. The final process is to move from the Course1 Object node to the HPolEnd node.

**Figure A4:**
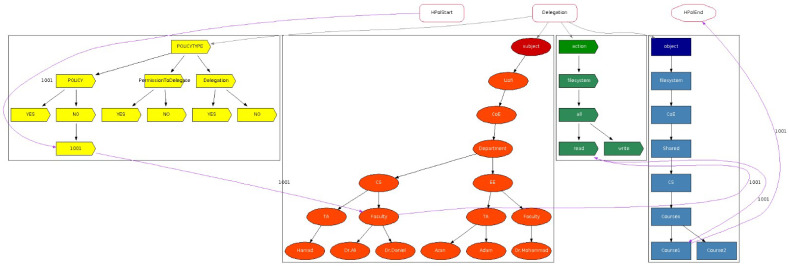
Resulting HPol model after requesting adding a negative policy to the empty HPol model example at the parent (subject) node. Yellow color represents policies. Orange color represents subjects. Green color represents action. Blue color represents objects.

On the other hand, in Figure A3, negative policies can be placed at different parent node levels, as shown in Figure A4. These policies follow the consistent path in the HPol model, beginning at HPolStart and concluding at HPolEnd. The purple arrow in Figure A3 represents that “CS” users are not allowed to read Course1. To establish this negative policy, it starts at HPolStart and proceeds to the newly created PPID node (PPID 1001), which connects to the “NO” node under the POLICY node. The path then goes from PPID 1001 to “CS”, then to the read Action, followed by Course1, and finally ends at HPolEnd. This configuration allows for diverse negative policies and aligns with permission delegation and delegation types covered in Section 3.5.3 and Section 3.5.6.

### 3.3. Permission to Delegate and Delegation in the HPol Model

HPol is a formal model for representing a system’s security and access control policies. It formalizes permissions using tuples (Who, What, Which) and employs directed acyclic graphs (DAGs) to connect (Subject, Action, Object) tuples, indicating allowed policies. Delegation is a key focus for organizational leaders. HPol addresses various aspects, including positive/negative permission to delegate and delegation. It helps businesses manage employee activities, such as file sharing and task delegation, bridging the gap between policy definition and implementation. Section 3.2 deals with permission to delegate, both positive and negative delegation, and incorporating positive/negative policies.

### 3.4. Research Method

In our research, we enhance the HPol model to address issues such as negative permissions, permission delegation (both positive and negative), and delegation, using layered Directed Acyclic Graphs (DAGs).

The HPol model employs a graph-based DAC approach with three subgraphs (DAGs) for subjects, actions, and objects. To tackle research challenges, we introduce a yellow subgraph connected to POLICYTYPE, containing nodes for (POLICY), (PermissionToDelegate), (Delegation), and WildCard, using “YES” for positive and “NO” for negative policies, creating a hierarchy. User policies are defined by nodes and edges, forming policy paths from HPolStart to HPolEnd. This approach applies to both positive and negative policies, permissions, and delegations, addressing hierarchy issues and negative permissions. For more details, refer to the Section 3.5.

### 3.5. Experiments

This section covers the implementation of permission delegation in the HPol model. It discusses positive and negative delegation, as well as enhancements to positive permission and the introduction of a new negative permission. Table 6 illustrates different combinations of policy, permission delegation, and delegation authorization, ranging from an empty policy (-) to “YES” or “NO” policy requests, “YES” or “NO” permission to delegate requests, and “YES” or “NO” delegation requests. Each experiment section provides step-by-step explanations and descriptions of the case studies.

In simple terms, (-) policy means a user has not requested permission, “YES” policy allows the user to read or write, and “NO” policy denies such access. “YES” permission to delegate allows delegation, while “NO” permission to delegate denies it. Positive delegation “YES” authorizes the delegation of permissions to another user, and negative delegation “NO” indicates that delegation is not possible due to a prior “NO” permission to delegate.

Our study shows that policies, permission delegation, positive delegation, and negative delegation can be effectively managed using the combinations in Table 6.

#### 3.5.1. Experiment: From Positive Policy (YES), Empty Permission to Delegate (-), and Empty Delegation (-) with an Add Positive Permission to Delegate (YES) Request

In this experiment, we explore the “YES” permission to delegate request in conjunction with a positive policy “YES”. We focus on the first option, describing the “YES” permission to delegate request, and the negative permission to delegate represented by “NO” in Experiments Section 3.5.5.

**Figure A5:**
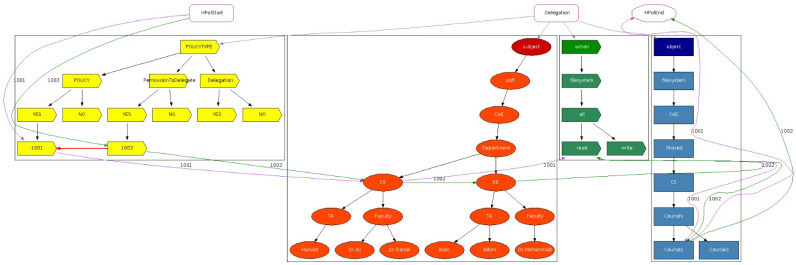
Resulting HPol model after requesting adding a positive permission to delegate (YES) at the grandparent (subject) node on a model with an existing positive policy (YES) at the grandparent (subject) node. Yellow color represents policies. Orange color represents subjects. Green color represents action. Blue color represents objects.

Table 7 outlines two different policy outcomes within the HPol model. We delve into the first scenario, where a user requests “YES” permission to delegate, already holding a positive policy “YES”, as depicted in Figure A2 to Figure A5, without any delegation requests yet.

In this initial scenario from Table 7, “CS” users permit a positive policy “YES” with PPID (1001) to read in Course1, represented by a purple arrow in Figure A2. Any node under the “CS” is subsequently granted a “YES” permission to delegate request to any node under the “EE” node through a green arrow with PPID (1002). The red link between PPID (1002) and (1001) clarifies the permissions available to delegate. The producer policy path steps have already been outlined in Experiments Section 3.2. The positive permission to delegate “YES” policy path request is therefore described in the following way:

1. The HPol model process typically begins at the HPolStart node and goes to the PPID (1002) node in the permission to delegate (DAG), which is under the “YES” permission to delegate node, as shown in Figure A5, indicating that “CS” users have the positive permission to delegate their policy to “EE” represented by a green arrow.
2. Move from the PPID (1002) node to the “CS” Subject node within the Subject (DAG).
3. Move from the “CS” Subject node to the “EE” Subject node within the Subject (DAG).
4. Move from the “EE” Subject node, to the read node in the Action (DAG).
5. From the read Action node, move to the Course1 node in the Objects (DAG).
6. The HPolEnd node, which must be connected, represents the endpoint of the HPol model. The last step is to move from the Course1 Object node to the HPolEnd node.

In the HPol model (Figure A2), “CS” members have a positive policy “YES” in the grandparent node, but only faculty has the positive permission to delegate “YES” to “TA” in the parent nodes. Both positive policy and permission to delegate “YES” paths use the same producer steps from Experiments Section 3.5.1. The distinction is their positions within the HPol model, allowing customized permission assignment, with the positive policy “YES” and permission to delegate request “YES” at different levels between parent and grandparent nodes.

**Figure A6:**
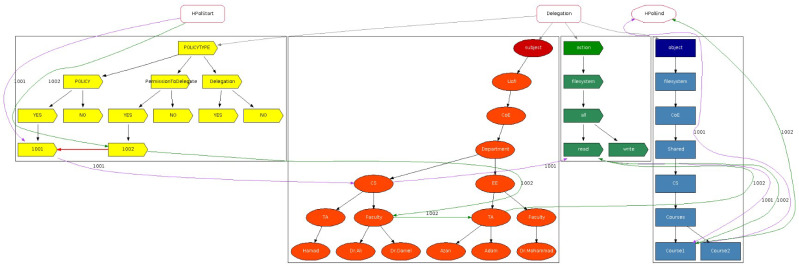
Resulting HPol model after requesting adding a positive permission to delegate (YES) at the parent (subject) node on a model with existing positive policy (YES) at the grandparent (subject) node. Yellow color represents policies. Orange color represents subjects. Green color represents action. Blue color represents objects.

Another scenario involves the positive policy “YES” and permission to delegate “YES” request in different level nodes, as displayed in Figure A6. This distinction between Figure A5 and Figure A6 allows for different levels of delegation requests, as explained in Experiments Section 3.5.4.

#### 3.5.2. Experiment: From Positive Policy (YES), Empty Permission to Delegate (-), and Empty Delegation (-) with an Add Negative Permission to Delegate (NO) Request

**Figure A7:**
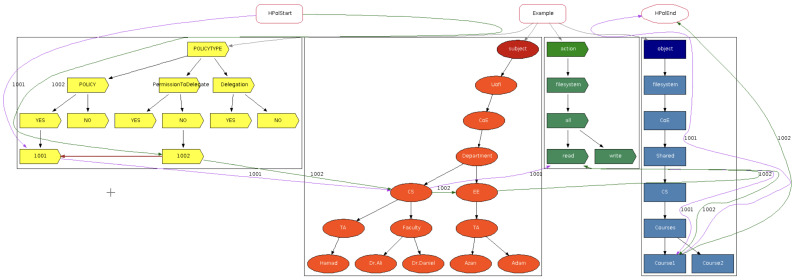
Resulting HPol model after requesting adding a negative permission to delegate (NO) at the grandparent (subject) node on a model with an existing positive policy (YES) at the grandparent (subject) node. Yellow color represents policies. Orange color represents subjects. Green color represents action. Blue color represents objects.

**Figure A8:**
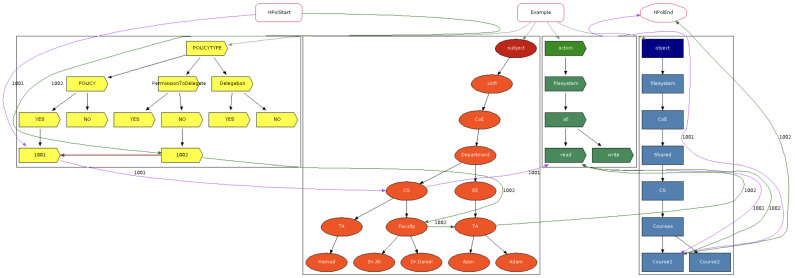
Resulting HPol model after requesting adding a negative permission to delegate (NO) at the parent (subject) node on a model with an existing positive policy (YES) at the grandparent (subject) node. Yellow color represents policies. Orange color represents subjects. Green color represents action. Blue color represents objects.

This experiment also takes a new approach to positive policy “YES”, and negative permission to delegate “NO” request. In Experiments Section 3.5.1, we described the “YES” option for permission to delegate with a positive policy, and in this experiment, we explain the other option, which is the negative permission to delegate “NO” request to the user even if the same user has a positive policy “YES”. Table 8 illustrates two scenarios for requesting negative permission to delegate “NO” in different level (Subject) nodes, as shown in Figure A2 to Figure A7 and Figure A2 to Figure A8.

The first scenario describes the current positive policy “YES” at the “CS” grandparent level (Subject) node, showing that anyone who has a positive policy “YES” can have an action read in Course1 in the object with a unique PPID (1001) produced. Furthermore, while having a positive policy “YES”, the request permission to delegate is “NO” at the grandparent (Subject) level node, implying that the “CS” users have negative permission to delegate “NO” to “EE” users with a unique PPID (1002) produced. A red link between the (1002) and (1001) PPID describing the wildcard indicates for which policy the user has the negative permission to delegate “NO”. The negative permission to delegate “NO” policy path request is therefore described in the following way:

1. The HPol model process typically begins at the HPolStart node and goes to the PPID (1002) node in the permission to delegate (DAG), which is under the “NO” permission to delegate node, as shown in Figure A7 with the green arrow, indicating the negative permission to delegate “NO” from the “CS” users to the “EE”.
2. Navigate from “CS” to “EE” (Subject) node within the Subject (DAG) from the PPID (1002) node.
3. From the EE Subject node, move to the read node in the Action (DAG).
4. From the read Action node, move to the Course1 node in the Objects (DAG).
5. The HPolEnd node, which must be connected, represents the endpoint of the HPol model. The last step is to move from the Course1 Object node to the HPolEnd node.

On the other hand, the positive policy “YES” can be authorized and established at the “CS” grandparent (Subject) level node while the negative permission to delegate “NO” request is requested at the Faculty parent (Subject) level node, as shown in Figure A8, which shows the result of the HPol model requesting negative permission to delegate “NO” at the parent (Subject) level node while an existing positive policy “YES” is in place at the “CS” grandparent (Subject) level node. Each policy has a unique PPID, such as (1001) for positive policy “YES”, (1002) for negative permission to delegate “NO”. This demonstrates that the “CS” users have the positive policy “YES” and the negative permission to delegate a “NO” request from the Faculty node under the CS node to the TA under the EE node, as shown in Figure A8.

So, in simple terms, the experiment definition is that a user has a “YES” positive policy but can not have a permission to delegate that policy to another user.

#### 3.5.3. Experiment: From Negative Policy (NO), Empty Permission to Delegate (-), and Empty Delegation (-) with an Add Negative Permission to Delegate (NO) Request

In this experiment, we study negative permission to delegate “NO” requests in the presence of existing negative policies “NO” at different levels.

**Figure A9:**
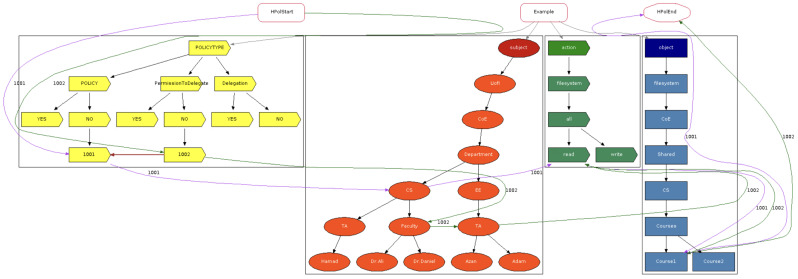
Resulting HPol model after requesting adding a negative permission to delegate (NO) at the parent (subject) node with an existing negative policy at the grandparent (subject) node. Yellow color represents policies. Orange color represents subjects. Green color represents action. Blue color represents objects.

In the first case of Table 9 (Figure A3, Figure A4, Figure A5, Figure A6, Figure A7, Figure A8 and Figure A9), we examine a negative permission to delegate “NO” request at the parent node with an existing negative policy at the grandparent CS node (Figure A3). Here, requesting positive permission to delegate “YES” at the same or lower levels is not permitted for three key reasons: (1) The existing negative policy mandates only a “NO” for the permission to delegate request, as resolved in this experiment. (2) It reveals a conflict between the current negative permission to delegate “NO” and a positive permission to delegate “YES” request, a topic for future consideration. (3) Approving positive permission to delegate “YES” requires addressing policy removal and editing, a future strategy that considers the existing policy type to avoid conflicts and redundancies.

We have resolved conflicts, redundancies, and partial policy requests involving only positive “YES” or negative “NO” policies in the publication under review [18]. As a result, permission to delegate requests must be exclusive of the negative type “NO” when there is an existing negative policy “NO”. However, if the request is for positive permission to delegate “YES” and there is an existing positive policy “YES”, it is considered a valid request, addressed in Experiments Section 3.5.1. To prevent conflicts between the current permission to delegate “NO” and the request for permission to delegate “YES”, we must simultaneously plan for policy removal and editing.

To illustrate a negative permission to delegate “NO” request, we consider the existing negative policy “NO” at the grandparent CS node, as shown in Figure A3 and Figure A9, which show the negative permission to delegate “NO” request in the parent node from Faculty under the CS node to the TA under the EE (Subject) node with a new unique PPID (1002). The process for the current negative policy “NO” and permission to delegate “NO” request follows similar steps as in a previous section, with the difference of the negative policy at the grandparent level (CS node) and the permission to delegate request at the parent level. The negative permission to delegate “NO” policy path request is therefore described in the following way:

1. The HPol model begins at the HPolStart node and moves to node PPID (1002) under the “NO” permission to delegate, as shown in Figure A9. The green arrow indicates negative permission to delegate “NO”, and a red link between PPID (1002) and PPID (1001) shows the specific policy with negative delegation permission “NO”.
2. Navigate from the Faculty to the TA Subject node within the Subject (DAG) from the PPID (1002) node.
3. Move from the TA Subject node to the read node in the Action (DAG).
4. Move from the read Action node to the Course1 node in the Objects (DAG).
5. The HPolEnd node, which must be connected, represents the endpoint of the HPol model. The last step is to move from the Course1 Object node to the HPolEnd node.

**Figure A10:**
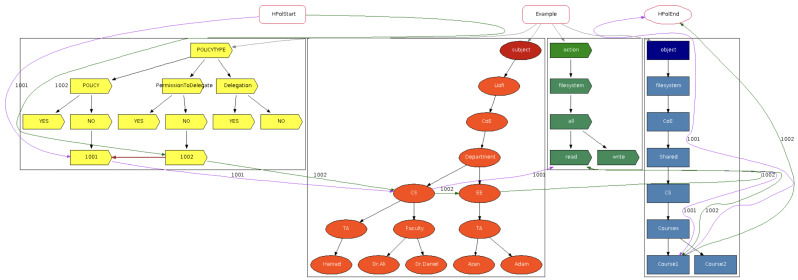
Resulting HPol model after requesting adding a negative permission to delegate (NO) at the grandparent (subject) node with an existing negative policy at the grandparent (subject) node. Yellow color represents policies. Orange color represents subjects. Green color represents action. Blue color represents objects.

On the other hand, for a different case study result approach, such as the second case, as stated in Table 9 from Figure A3, the present negative policy “NO” is described first at the grandparent (Subject) level node, and Figure A10 demonstrates the negative permission to delegate “NO” request at the grandparent (Subject) level node too.

Figure A9 and Figure A10 show the current policy at a different level and the request policy at the same or other different level. Figure A9 describes the negative policy “NO” in the grandparent node and the permission to delegate “NO” request in the parent node, whereas Figure A10 describes the negative policy “NO” in the grandparent node and the permission to delegate “NO” request in the grandparent node.

#### 3.5.4. Experiment: From Positive Policy (YES), Permission to Delegate (YES), and Empty Delegation (-) with an Add Positive Delegation (YES) Request

In this experiment, we explore positive delegation “YES”, impacting the case study results in the HPol model (Table 10). Delegation has two options: approval “YES” and rejection “NO”. This section covers positive delegation, while negative delegation is discussed in Experiments Section 3.5.5 and Section 3.5.6, which rely on the “NO” permission to delegate policy.

Positive policy “YES” explains how policies are created and assigned to a user. A unique PPID node links to the “YES” node under the (Policy) node for positive policies. This approach is similar to permission to delegate and delegation but applies to specific nodes under permission to delegate or delegation.

**Figure A11:**
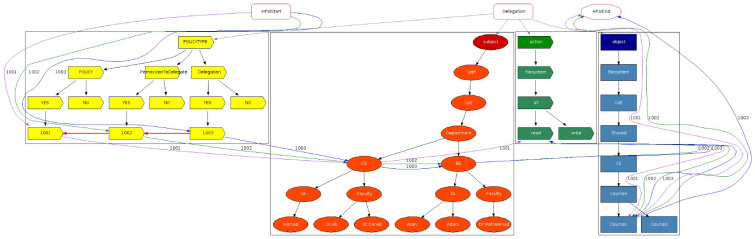
Resulting HPol model after requesting adding a positive delegation (YES) at the grandparent (subject) node on a model with existing positive policy (YES) and positive permission to delegate (YES) at the grandparent (subject) node. Yellow color represents policies. Orange color represents subjects. Green color represents action. Blue color represents objects.

In the first case, as illustrated in Table 10 from Figure A5 to Figure A11, users under “CS” have a positive policy “YES” at the grandparent (Subject) node, permission to delegate “YES” at the grandparent (Subject) node, and a positive delegation “YES” request, certifying CS’s users permission to read a file and delegate that policy with a positive delegation request “YES” to another user. Figure A11 showcases the implementation of Experiment Section 3.5.4 as a case study, including positive policy, permission to delegate, and positive delegation. The positive delegation “YES” policy path request is therefore described in the following way:

1. The HPol model starts at HPolStart and proceeds to PPID (1003) in the positive delegation (DAG) under the “YES” delegation node, as indicated by the blue link in Figure A11, showing CS’s users have the delegation permission of the policy to EE users. The red arrow with a wildcard signifies the connection policy between PPID (1003) and PPID (1002) for CS’s positive delegation and permission to delegate.
2. Move from the PPID (1003) node to the CS Subject node within the Subject (DAG).
3. Navigate to the CS from the EE Subject node within the Subject (DAG).
4. Move from the EE Subject node to the read Action node within the Action (DAG).
5. From the read Action node, move to the Course1 node in the Objects (DAG).
6. From the Course1 Object node, move to the HPolEnd node.

**Figure A12:**
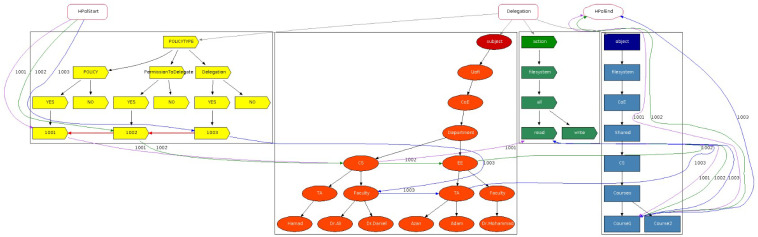
Resulting HPol model after requesting adding a positive delegation (YES) at the parent (subject) node on a model with existing positive policy (YES) and positive permission to delegate (YES) at the grandparent (subject) node. Yellow color represents policies. Orange color represents subjects. Green color represents action. Blue color represents objects.

In a different scenario (Table 10), Figure A5, Figure A6, Figure A7, Figure A8, Figure A9, Figure A10, Figure A11 and Figure A12 show a unique positive delegation “YES” request. Figure A5 displays the current “YES” policy at the “CS” grandparent level, with faculty node delegation of “YES” to TA at the parent level node. In Figure A12, faculty’s request for positive delegation “YES” to TA node at the parent level is highlighted. Importantly, only faculty node under the CS node, a faculty member, can grant delegation permission to the teaching assistant TA under the EE node. The process aligns with Experiments Section 3.5.4, but it differs in the policy and delegation levels, focusing on a positive delegation “YES” request at the parent level node in the hierarchy structure.

**Figure A13:**
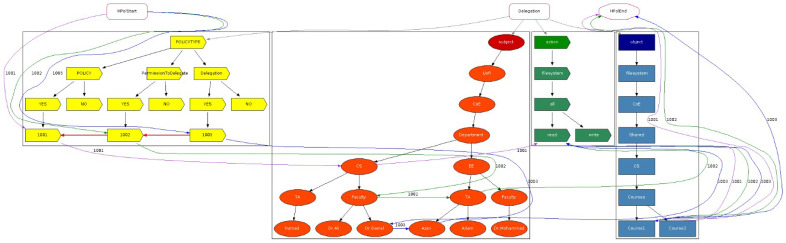
Resulting HPol model after requesting adding a positive delegation (YES) at the child (subject) node on a model with an existing positive policy (YES) at the grandparent (subject) node and positive permission to delegate (YES) at the parent (subject) node. Yellow color represents policies. Orange color represents subjects. Green color represents action. Blue color represents objects.

Moreover, Table 10 details the final case study, including the existing policy “YES”, positive delegation permission “YES”, and a positive delegation “YES” request across various (Subject) level nodes, as shown in Figure A5 to Figure A13. In Figure A13, a positive delegation request occurs at a different level, with a pre-existing positive policy at the grandparent CS node, current delegation permission at the Faculty parent node, and the request for positive delegation “YES” at the child node. Dr. Daniel delegates to Azan, who is a teaching assistant TA under the EE node.

#### 3.5.5. Experiment: From Positive Policy (YES), Negative Permission to Delegate (NO), and Empty Delegation (-) with an Add Negative Delegation (NO) Request

In this experiment, we explore an alternative approach with positive policies “YES”, negative delegation requests “NO”, and negative permissions to delegate “NO”.

**Figure A14:**
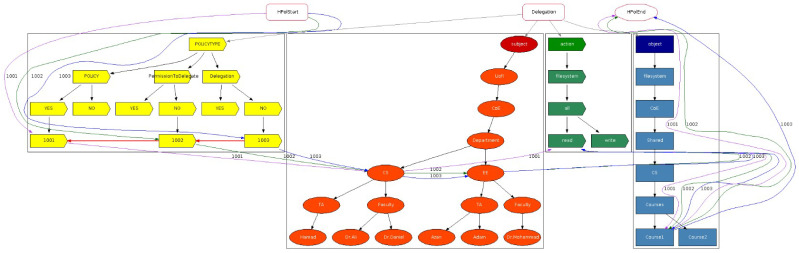
Resulting HPol model after requesting adding a negative delegation (NO) at the same (subject) node on a model with an existing positive policy (YES) and negative permission to delegate (NO) at the same (subject) node. Yellow color represents policies. Orange color represents subjects. Green color represents action. Blue color represents objects.

**Figure A15:**
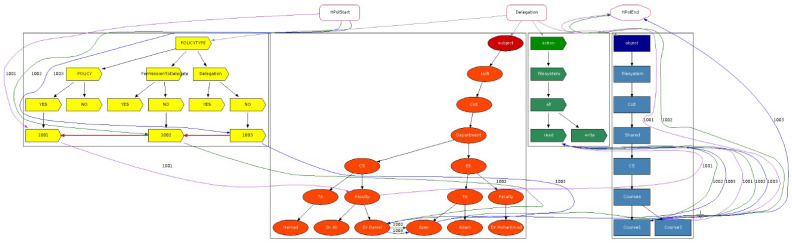
Resulting HPol model after requesting adding a negative delegation (NO) at the child (subject) node on a model with an existing positive policy (YES) at the parent (subject) node and negative permission to delegate (NO) at the child (subject) node. Yellow color represents policies. Orange color represents subjects. Green color represents action. Blue color represents objects.

Table 11 shows two scenarios for negative delegation requests at different (Subject) level nodes, going from Figure A7 to Figure A14 and from Figure A8 to Figure A15.

The first scenario involves a positive policy “YES” at the grandparent level (Subject) node, indicating that all “CS” members have a positive policy “YES” for reading Course1. However, in the presence of current negative permissions to delegate “NO”, any delegation request must be negative “NO” to comply with the rules in Section 3.6.4. We will now describe the policy path for the negative delegation “NO” request within the HPol model. The negative delegation “NO” policy path request is therefore described in the following way:

1. The HPol model process is primarily initiated at the HPolStart node and moves to the PPID (1003) node in the negative delegation (DAG), which is under the “NO” delegation node, as shown in Figure A14, signifying that all CS users are unauthorized to delegate to the user EE users node. The red arrow describing the wildcard indicates the connection policy between the (1003) and (1002) PPID regarding which policy has the following negative delegation.
2. Navigate from the CS to EE Subject node within the Subject (DAG) from the PPID (1003) node.
3. Move from the EE Subject node to the read Action node within the Action (DAG).
4. From the read Action node, move to the Course1 node in the Objects (DAG).
5. From the Course1 Object node, move to the HPolEnd node.

On the other side, positive policies “YES” and negative permissions to delegate “NO” can be established at a different Subject level node, as dictated by the policy rule in Section 3.6.4. Refer to Figure A15, which shows the HPol model requesting negative delegation “NO” at the same Subject level node where a positive policy “YES” and permission to delegate also exist. Each policy has a unique PPID: (1001) for the current positive policy “YES”, (1002) for the current negative permission to delegate “NO”, and (1003) for the request negative delegation “NO”.

#### 3.5.6. Experiment: From Negative Policy (NO), Negative Permission to Delegate (NO), and Empty Delegation (-) with an Add Negative Delegation (NO) Request

In Table 12, we examine case studies involving the current negative policy “NO”, current negative permission to delegate “NO”, and negative delegation “NO” requests in the HPol model.

**Figure A16:**
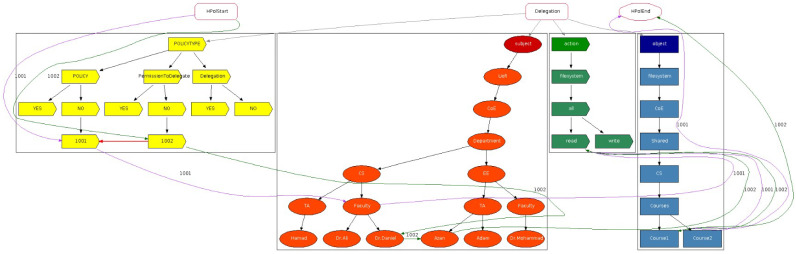
Resulting HPol model after requesting adding a negative permission to delegate (NO) at the child (subject) node on a model with an existing negative policy (NO) at the parent (subject) node. Yellow color represents policies. Orange color represents subjects. Green color represents action. Blue color represents objects.

**Figure A17:**
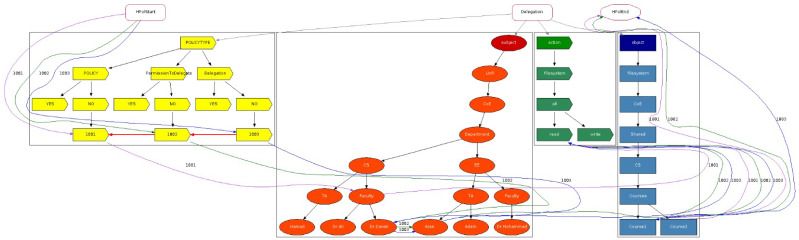
Resulting HPol model after requesting adding a negative delegation (NO) at the child (subject) node on a model with an existing negative policy (NO) at the parent (subject) node and negative permission to delegate (NO) at the child (subject) node. Yellow color represents policies. Orange color represents subjects. Green color represents action. Blue color represents objects.

In the first case, from Figure A16 to Figure A17, we explore a negative delegation “NO” request at a child node, considering the parent’s current negative policy “NO” and the child’s negative permission to delegate “NO”. This requires a negative delegation “NO” request, as specified in Section 3.6.4. To summarize the policy paths for the current negative policy “NO” and negative permission to delegate “NO”, we refer to Experiments Section 3.5.3. The negative delegation “NO” policy path request is therefore described in the following way:

1. The HPol model process is primarily initiated at the HPolStart node and moves to the PPID (1003) node in the negative delegation (DAG), which is under the “NO” delegation node, as depicted in Figure A17, signifying that the Dr. Daniel user denies to delegate to the user Azan. The red arrow, represented by a wildcard, signifies the connection policy between (1003) and (1002) PPID for determining the negative delegation.
2. Navigate from the Dr. Daniel to Azan Subject node within the Subject (DAG) from the PPID (1003) node.
3. Move from the Azan Subject node to the read Action node within the Action (DAG).
4. From the read Action node, move to the Course1 node in the Objects (DAG).
5. From the Course1 Object node, move to the HPolEnd node.

**Figure A18:**
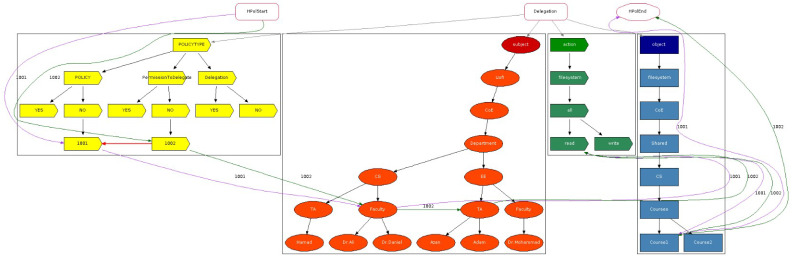
Resulting HPol model after requesting adding a negative permission to delegate (NO) at the parent (subject) node on a model with an existing negative policy (NO) at the parent (subject) node. Yellow color represents policies. Orange color represents subjects. Green color represents action. Blue color represents objects.

**Figure A19:**
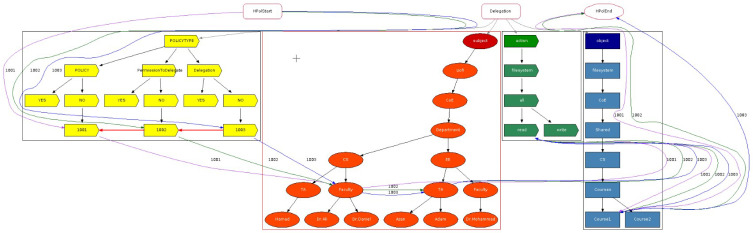
Resulting HPol model after requesting adding a negative delegation (NO) at the parent (subject) node on a model with an existing negative policy (NO) at the parent (subject) node and negative permission to delegate (NO) at the parent (subject) node. Yellow color represents policies. Orange color represents subjects. Green color represents action. Blue color represents objects.

In various case studies, we observe different approaches to the request for negative delegation “NO”, as seen in the second case detailed in Table 12 (from Figure A18 to Figure A19). Figure A18 depicts the current negative policy “NO” and the current negative permission to delegate “NO” at the same (Subject) level node. Figure A19 illustrates a negative delegation “NO” request at the same (Subject) level node.

Figure A17 and Figure A19 in the HPol model differ because Figure A17 illustrates a negative delegation “NO” request at the child (Subject) level node, with an existing negative policy at the parent (Subject) level node and negative permission to delegate “NO” at the child (Subject) level node. In contrast, Figure A19 shows a negative delegation “NO” request at the same (Subject) level node, with existing negative policy and negative permission to delegate “NO” at that level.

### 3.6. Policy Rules Request Based on HPol Model

A set of rules is required in the field of delegation and permissions management to ensure the orderly and secure transfer of duties and access within an organization. These rules are intended to protect the delegation process’s integrity and transparency by requiring it to follow preset policies and permissions. We provide four fundamental rules for policies, permission to delegate, and delegation.

#### 3.6.1. Rule: To Request Permission to Delegate, a Corresponding Policy Must Be Predefined

This rule covers two types of delegation requests: positive (YES) and negative (NO), each with predefined policies. Before granting delegation permissions, a policy must be in place, specifying who can delegate, what can be delegated, and when. For instance, Figure A5, Figure A6, Figure A7, Figure A8, Figure A9 and Figure A10 demonstrate the permission to delegate request with their respective policies, showing where the request can originate within the hierarchy.

#### 3.6.2. Rule: To Request a Delegation, a Corresponding Permission to Delegate Must Be Predefined

This rule applies to two delegation types: positive (YES) and negative (NO), each with a predefined permission policy. Users must have relevant delegation permission before performing delegation, a common principle in access control and authorization systems. This ensures regulated and authorized delegation, maintaining security and control. Figure A11, Figure A12, Figure A13, Figure A14, Figure A15, Figure A17 and Figure A19 illustrate how predefined permission to delegate policies dictate positive or negative delegation in different hierarchy levels, ensuring traceability and accountability.

#### 3.6.3. Rule: If a Negative Policy (NO) Is Defined, the Permission to Delegate Request Must Be Negative (NO)

This rule dictates that when a policy restricts actions, delegation permissions must also be restricted, a common practice in access control systems to maintain system integrity and security by preventing users from bypassing their restrictions. Figure A9 and Figure A10 illustrate that permission to delegate requests must be negative (NO) when a user has a negative policy (NO). Granting positive delegation (YES) to such users is a security breach, requiring policy exception, removal or modification.

#### 3.6.4. Rule: If a Negative Permission to Delegate (NO) Is Defined, the Delegation Request Must Also Be Negative (NO)

This rule suggests that if someone’s current permission to delegate is negative, then any ongoing or future delegation of tasks or responsibilities by that individual should also be negative. In other words, permission to delegate and the actual act of delegation should be aligned. For example, Figure A14, Figure A15, Figure A17 and Figure A19 highlight specific restrictions, such as the negative (NO) of a delegation request when the permission to delegate is set as a negative policy.

## 4. Conclusions

The article explores advancements in the HPol model, aiming to address its limitations and align with contemporary access control models. It focuses on enhancing support for permission to delegate and delegation, covering both positive and negative scenarios. Automated checks ensure corresponding policies are in place before granting permissions.

Improvements to the HPol formal model and Python code implementation are made, accompanied by an analysis of various scenarios and case studies. These enhancements empower system developers and security professionals to create more secure systems and embrace zero-trust architectures. In addition to this, by improving the transparency, control, and distributed security, the model recommended establishes a structure for blockchain-integrated delegation policies in intelligent Internet of Things (IoT) systems.

## 5. Future Work

Using the delegation model as a blockchain-based access control system is a useful potential approach. It is possible to make permission to delegate enforcement decentralized and verifiable with the help of smart contracts. In environments where trust limitations are changing and auditability is needed, such as smart cities, autonomous systems, or Industrial IoT, this would allow for secure delegation. It is critical to automatically check for conflicts and violations between the positive and negative policy or delegation with the separation of duties policies. We are updating HPol to fully support SoD rules functionality, which includes SoD types, redundancy, conflict detection within the SoD policy itself, and SoD violation. We plan to disclose our work in progress in an upcoming article. The work presented here is formalized through the use of graph modeling. In the future, we intend to describe the model using a formal language. In the interest of timely publication and comprehensibility of our work, we decided to leave additional formalization for future articles. In addition to this, we intend to test the security, privacy, scalability, and runtime performance aspects of our model and other related work items in a future article. 

## Figures and Tables

**Table 1:** Descriptions of access control challenges in permission to delegate and delegation redundancy and conflict detection policies.

Challenge Number	Challenge Name		Description	Our Model Support
1	Positive and Negative Permission to Delegate Plus Redundancy and Conflict Detection	Positive Permission to Delegate	Describes the process of allowing individuals in an organization to delegate tasks, responsibilities, or decision making authority to others constructively and effectively.	✓
		Negative Permission to Delegate	Represents the methods of preventing a user or users in an organization from successfully delegating a variety of tasks, duties, or decision-making authority to others.	✓
		Positive Permission to Delegate Policy Redundancy	Refers to the systematic duplication or repetition of policies that grant authority and promote the practice of delegating duties and responsibilities.	✕
		Positive Permission to Delegate Policy Conflict	Refers to the situation where two separate policies exist to authorize and regulate delegation at the same or different levels of the hierarchy, leading to policy inconsistencies or confusion between users.	✕
		Negative Permission to Delegate Policy Redundancy	Indicates that the same policy exists at the same or different levels of the hierarchy policy structure.	✕
		Negative Permission to Delegate Policy Conflict	This term refers to a situation within an organization in which there is positive and negative permission to delegate at the same or a different level in the hierarchy structure policy at the same time.	✕
2	Positive and Negative Delegation Policy Plus Redundancy and Conflict Detection	Positive Delegation Policy	Positive delegation is the act of delegating a task responsibility to another user or group.	✓
		Negative Delegation Policy	Describes policy that prohibits the user or users from delegating actions to others.	✓
		Positive Delegation Policy Redundancy	Describes policy duplication that grants authority and promotes the practice of assigning duties and responsibilities at the same or various levels in a hierarchy structure.	✕
		Negative Delegation Policy Redundancy	Describes policy redundancy, which is the denial of authority when delegating tasks and duties at the same or different levels of a hierarchy structure.	✕
		Positive Delegation Policy Conflict	Conflicting delegation policies occur when positive and negative delegation policies occur at the same or different levels of the hierarchy structure.	✕
		Negative Delegation Policy Conflict	When two different policies, such as positive and negative policies, emerge at the same or separate levels of the hierarchy at the same time, this is referred to as conflicting policy.	✕

**Table 2:** Attributes of delegation.

Characteristic Attribute	Representative Features
Who is requesting a delegation?	- User—System Authority
Who will be the delegator?	- User—System Authority (Administrative)
How much can I delegate?	- Total—Partial
How long do you delegate for?	- Temporary—Permanent
How do we delegate?	- Grant—Transfer
Where will the delegation take place?	- User Level—System Level

**Table 3:** Detail of HPol models used to describe the starting model and resulting model for experiments. From empty policy (-) to positive policy (YES), and from empty policy to negative policy (NO).

	Figure	Model	Policy
From	Figure A1	Empty Policy	-
To	Figure A2	Positive Policy	YES
From	Figure A1	Empty Policy	-
To	Figure A3	Negative Policy	NO

**Table 4:** Detail of starting model and resulting model for experiment: From empty policy (-) model to positive policy model (YES) request.

	Figure	Model	Policy
From	Figure A1	Empty Policy	**-**
To	Figure A2	Positive Policy	YES

**Table 5:** Detail of starting model and resulting model for experiment: From empty policy (-) model to negative policy model (NO) request.

	Figure	Model	Policy
From	Figure A1	Empty Policy	-
To	Figure A3 and Figure A4	Negative Policy	NO

**Table 6:** Detail of valid HPol models used to describe the starting model and resulting model after considering policy, permission to delegate, and delegation and their potential interactions.

Figure	Policy	Permission to Delegate	Delegation
Figure A1	-	-	-
Figure A2	YES	-	-
Figure A3 and Figure A4	NO	-	-
Figure A5 and Figure A6	YES	YES	-
Figure A7 and Figure A8	YES	NO	-
Figure A9, Figure A10, Figure A16 and Figure A18	NO	NO	-
Figure A11, Figure A12 and Figure A13	YES	YES	YES
Figure A14 and Figure A15	YES	NO	NO
Figure A17 and Figure A19	NO	NO	NO

**Table 7:** Detail of starting model and resulting model considering policy, permission to delegate, and delegation for experiment: From positive policy (YES), empty permission to delegate (-), and empty delegation (-) with an add positive permission to delegate (YES) request.

	Figure	Policy	Permission to Delegate	Delegation
From	Figure A2	YES	-	-
To	Figure A5	YES	YES	-
From	Figure A2	YES	-	-
To	Figure A6	YES	YES	-

**Table 8:** Detail of starting model and resulting model considering policy, permission to delegate, and delegation for experiments: From positive policy (YES), empty permission to delegate (-), and empty delegation (-) with an add negative permission to delegate (NO) request.

	Figure	Policy	Permission to Delegate	Delegation
From	Figure A2	YES	-	-
To	Figure A7	YES	NO	-
From	Figure A2	YES	-	-
To	Figure A8	YES	NO	-

**Table 9:** Detail of starting model and resulting model considering policy, permission to delegate, and delegation for experiment: From negative policy (NO), empty permission to delegate (-), and empty delegation (-) with an add negative permission to delegate (NO) request.

	Figure	Policy	Permission to Delegate	Delegation
From	Figure A3	NO	-	-
To	Figure A9	NO	NO	-
From	Figure A3	NO	-	-
To	Figure A10	NO	NO	-

**Table 10:** Detail of starting model and resulting model considering policy, permission to delegate, and delegation for experiment: From positive policy (YES), permission to delegate (YES), and empty delegation (-) with an add positive delegation (YES) request.

	Figure	Policy	Permission to Delegate	Delegation
From	Figure A5	YES	YES	-
To	Figure A11	YES	YES	YES
From	Figure A5	YES	YES	-
To	Figure A12	YES	YES	YES
From	Figure A5	YES	YES	-
To	Figure A13	YES	YES	YES

**Table 11:** Detail of starting model and resulting model considering policy, permission to delegate, and delegation for experiments: From positive policy (YES), negative permission to delegate (NO), and empty delegation (-) with an add negative delegation (NO) request.

	Figure	Policy	Permission to Delegate	Delegation
From	Figure A7	YES	NO	-
To	Figure A14	YES	NO	NO
From	Figure A8	YES	NO	-
To	Figure A15	YES	NO	NO

**Table 12:** Detail of starting model and resulting model considering policy, permission to delegate, and delegation for experiment: From negative policy (NO), negative permission to delegate (NO), and empty delegation (-) with an add negative delegation (NO) request.

	Figure	Policy	Permission to Delegate	Delegation
From	Figure A16	NO	NO	-
To	Figure A17	NO	NO	NO
From	Figure A18	NO	NO	-
To	Figure A19	NO	NO	NO

## Data Availability

No new data were created or analyzed in this study.
